# Realizing total reciprocity violation in the phase for photon scattering

**DOI:** 10.1038/srep43114

**Published:** 2017-02-22

**Authors:** László Deák, László Bottyán, Tamás Fülöp, Dániel Géza Merkel, Dénes Lajos Nagy, Szilárd Sajti, Kai Sven Schulze, Hartmut Spiering, Ingo Uschmann, Hans-Christian Wille

**Affiliations:** 1 Wigner RCP, RMKI, P.O.B. 49, 1525 Budapest, Hungary; 2 Budapest University of Technology and Economics, 3 Műegyetem rkp., 1111 Budapest, Hungary; 3 European Synchrotron Radiation Facility, BP 220, 38043 Grenoble, France; 4 Helmholtz-Institut Jena, Fröbelstieg 3, 07743 Jena, Germany; 5 Friedrich-Schiller-Universität Jena, Max-Wien-Platz 1, 07743 Jena, Germany; 6 Johannes Gutenberg Universität Mainz, Staudinger Weg 9, 55099 Mainz, Germany; 7 Deutsches Elektronen-Synchrotron (PETRA III), Notkestrasse 85, 22607 Hamburg, Germany

## Abstract

Reciprocity is when wave or quantum scattering satisfies a symmetry property, connecting a scattering process with the reversed one. While reciprocity involves the interchange of source and detector, it is fundamentally different from rotational invariance, and is a generalization of time reversal invariance, occurring in absorptive media as well. Due to its presence at diverse areas of physics, it admits a wide variety of applications. For polarization dependent scatterings, reciprocity is often violated, but violation in the phase of the scattering amplitude is much harder to experimentally observe than violation in magnitude. Enabled by the advantageous properties of nuclear resonance scattering of synchrotron radiation, we have measured maximal, i.e., 180-degree, reciprocity violation in the phase. For accessing phase information, we introduced a new version of stroboscopic detection. The scattering setting was devised based on a generalized reciprocity theorem that opens the way to construct new types of reciprocity related devices.

Reciprocity appears as a principle in various forms in diverse fields of human thinking, from jurisprudence through game theory^1^ to physics. Its first known written form appeared in the Code of Hammurabi (c. 1754 BC) as the rule ‘An eye for an eye, a tooth for a tooth’, expressing the principle of reciprocal judgement. In his *Principia*, Newton postulated the action–reaction principle, manifestation of reciprocity in classical mechanics. The extension to linear classical waves, taking into account the finite speed of propagation, was given by Helmholtz^2^, in the form of the statement that the interchange of source and detector does not change the scattering amplitude of a wave scattering process. This principle, established 150 years ago, has since been studied for various types of waves in numerous fields of physics^3^.

For scalar waves, reciprocity always holds. For waves with spin or polarization components, such as for propagation of photons and of neutrons, a sufficient condition is that the scatterer potential or permittivity, permeability, conductivity tensor is a self-transpose matrix in the polarization degree of freedom^3^,^4^ – here, transposition corresponds to the interchange of outgoing polarization with the incoming one. Optically active and magneto-optical media are examples for non-self-transpose cases, enabling nonreciprocal wave propagation. Various conditions and limitations to the reciprocity principle have been derived [see ref. 3 and references therein], and diverse applications have been born in the field of local and nonlocal electromagnetism^2^,^5^,^6^, sound waves^7^, electric circuits^8^, radio communication^9^, local and nonlocal quantum mechanical scattering problems^10^,^11^ etc. Nonreciprocal devices (circulators and isolators) with on-chip integration possibility have also been suggested^12^. We note that, in X-ray optics, the term nonreciprocity may also refer to time-reversal odd optical activity^13^,^14^,^15^,^16^, a meaning differing from the Helmholtz-originated one considered here.

## Reciprocity violation: a way to make it visible

Our research presented here aimed at investigating that, in non-self-transpose cases of polarization dependent scattering, reciprocity may hold or may be violated. To study this, highly polarization dependent scattering phenomena are advantageous. Nuclear resonance scattering of photons^17^, chosen here, is such an excellent possibility since nuclear resonances are extremely intense and scattering depends strongly on the local magnetic field that induces polarization dependent scattering. Nevertheless, to ensure a strong enough source delivering appropriately polarized photons, applying synchrotron radiation is the feasible way. With such a setting, magnitude reciprocity – i.e., equality of the absolute values of scattering amplitudes – can indeed be experimentally checked via measuring intensities, that is, photon detector counts^18^. On the other side, the involved frequencies of X-ray photons mean oscillations in the attosecond range so phase information can be extracted only via some kind of interferometric method, like done in X-ray holography^19^. For nuclear resonant scattering, an available method, essentially a variant of LLL (triple Laue) interferometry^20^, is the *stroboscopic technique*^21^,^22^, where – either built in the detection or as a signal post-processing – a window function of a train of enabling–disabling intervals is applied.

Actually, one can observe that the stroboscopic technique with such a window function is related to the real part of the scattering amplitude. This gave us the hint that, via a suitably modified window function, the imaginary part could also be accessed. (See these details in the Supplementary Information, in Section S5). Finding such a window function indeed turned out to be possible, opening the way for obtaining the phase information.

Another constituent in studying violation of reciprocity in the phase is the choice of an appropriate scattering process. A salient example would be a one where magnitude reciprocity holds – so standard intensity measurements would not see any difference between the direct scattering process and the reversed one – but, in phase, violation is maximal, i.e., the phase difference is 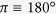. Unfortunately, the reciprocity theorem speaks only about *equality* of two certain scattering amplitudes and says nothing about when and how much two scattering amplitudes can be *nonequal*. What one can utilize to find a desired example is a recent generalization^4^ of the reciprocity theorem, which addressed the problem that self-transposeness is a polarization basis dependent notion, and extended the possibility to find a partner process to a given scattering process with the same scattering amplitude. Namely, self-transposeness turned out to be generalizable to the level where an arbitrary unitary operator connects the scattering amplitude with the reciprocal one. In such cases, for the partner process, the *direction* of propagation is still the reversed one, whereas the partner *polarizations* are not simply the reversed ones but are calculated from the unitary operator in question. This is the theoretical framework exploiting which a recent experiment succeeded in presenting large and tuneable reciprocity violation in magnitude^18^, for nuclear resonance scattering of synchrotron radiation on two ferromagnetic ^57^Fe absorber foils uniformly magnetized by permanent magnets. Switching magnitude reciprocity on and off was governed by changing the magnetization directions.

## Finding an appropriate setting

The concrete scattering arrangement used for our experiment has been devised as follows. In the case of coherent transmission through stratified media, the applicable generalized reciprocity condition simplifies to


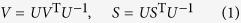


(see the Supplementary Information, Section S1), where the 2 × 2 matrix optical potential *V* describes dependence of the scattering on polarization^23^, *S* is the corresponding scattering (transmissivity) matrix^24^, ^T^ denotes the transpose, and *U* is an arbitrary unitary matrix.

In particular, in nuclear resonance forward scattering of synchrotron radiation on a ferromagnetic ^57^Fe sample – the case considered here –, six resonance lines are excited, pairwise corresponding to transitions with three different magnetic quantum number changes. Correspondingly, three different polarization dependent terms appear in the optical potential, proportional to


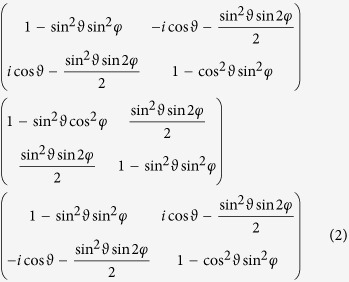


(see the Supplementary Information, Section S2), where *ϑ* and *φ* denote the polar angles of the magnetic field direction in the coordinate system distinguished by the synchrotron beam, **e**_*z*_ being the incident direction, 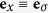 the horizontal (*σ*) polarization and 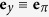 the vertical (*π*) polarization. Apparently, two of these matrices are not self-transpose in general. If one finds a common *U* with which each of these *V* s satisfies (1) then all the three scattering contributions behave the same way regarding reciprocity violation. This can be realized by


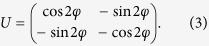


An inconvenience is that reciprocity speaks about interchange of source and detector but, obviously, one cannot interchange a whole synchrotron facility with the detector – the solution is to perform an appropriate rotation on the sample, instead. In polarization space, rotations act via another unitary matrix *U* ^r ^25, and (1) leads, for the rotated scattering matrix *S*^r^, to


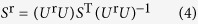


(Ref. 4, see also Section S2 of the Supplementary Information). The axis of the required 180 degree rotation can be taken freely in the **e**_*σ*_ − **e**_*π*_ plane. Denoting its angle with **e**_*σ*_ by *α*, one finds


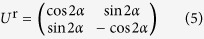


and, hence,


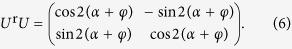


Both the experiment and the analysis are particularly simple if one chooses cos 2 (*α* + *φ*) = 0,


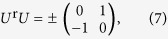


as the reversed-rotated scattering matrix elements are then related to those of the direct process as


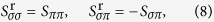



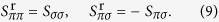


Since the coefficients −1 = exp(*iπ*) here mean a 180° phase shift between direct and reciprocal scattering matrices, a *σ* → *π* scattering process indeed provides an example where magnitudes are the same so magnitude reciprocity holds while, in phase, violation of reciprocity is maximal. This is the case we have realized, the concrete choices for magnetization and rotation angles being


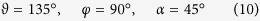


(see Fig. 1).

## Experiment and data analysis

**Figure 1:**
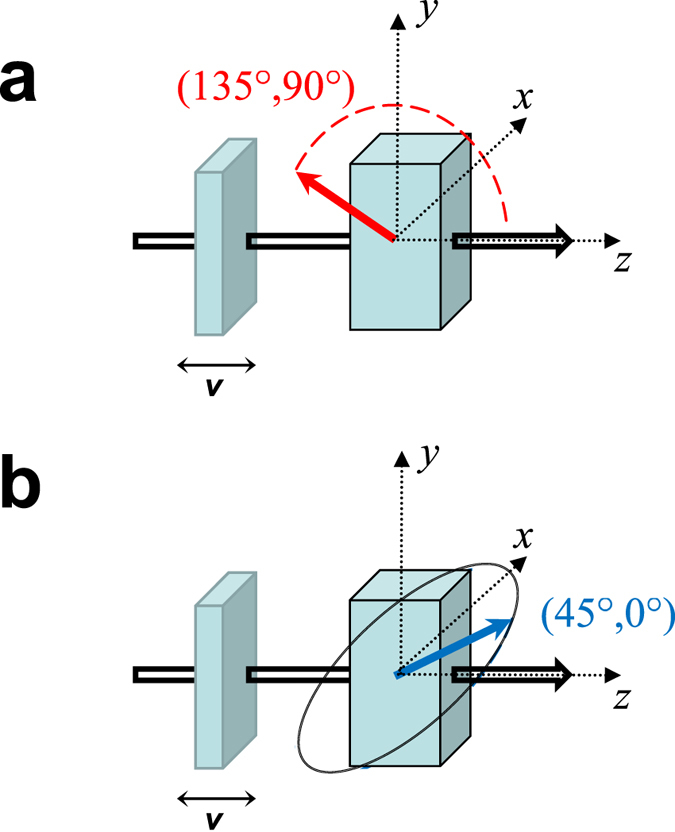
Coherent transmission through two stratified scatterers, arranged in a heterodyne setup. After a polarizer ensuring high-quality *σ* polarization, the beam arrived at a stainless steel sample (left) moved via a Mössbauer drive, and, subsequently, travelled through an *α*–^57^Fe foil (right). The coloured solid arrows depict the direction of magnetization of the latter sample in the direct (**a**) and reciprocal (**b**) scattering case, respectively. Rotation by 180° around the direction 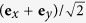 produced the reciprocal arrangement from the direct one. After the second scattering stage, the beam continued through an analyzer, and arrived at a detector.

**Figure 2:**
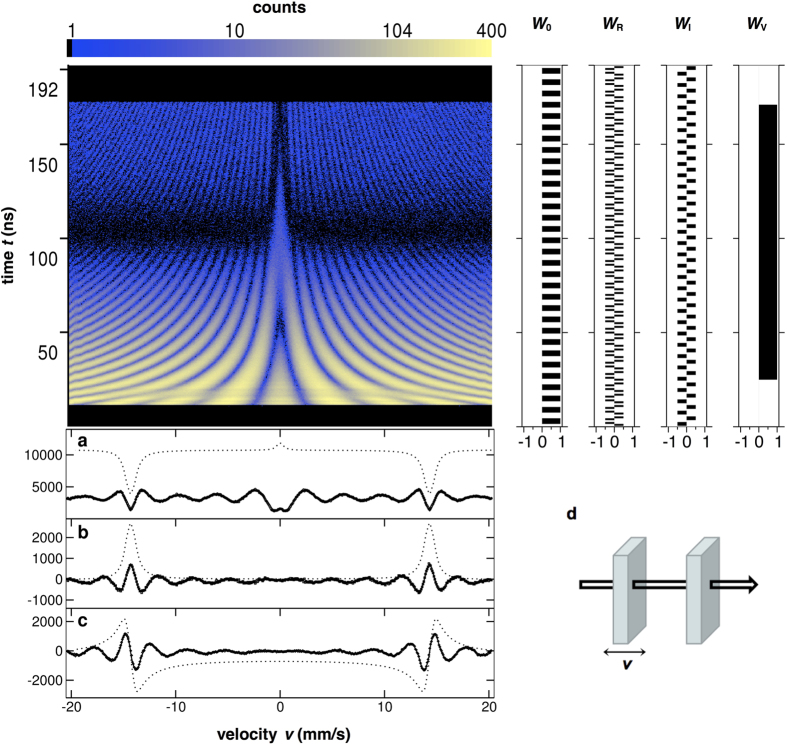
2D intensity pattern of nuclear resonant scattering of synchrotron radiation when calibrating the heterodyne setup. At this stage, two 2μm thick stainless steel ^57^Fe foils were placed in the heterodyne arrangement (**d**), one of them moved via a Mössbauer drive. From the measurement data, the drive and the foil width could be calibrated precisely. The 2D pattern has been evaluated by means of the stroboscopic approach, too. Time window *W*_0_ is that of classical stroboscopy^21^, *W*_R_ is an enhanced variant (minimizing overlaps of resonances) used for the real part of the scattering amplitude, and *W*_I_ is introduced for the imaginary part (and optimized similarly). Experimental conditions added some vetoes (disabled certain time intervals) as shown via *W*_V_. (**a**–**c**) Display the analysed signals (experimental and fitted) using time windows *W*_*V*_*W*_0_, *W*_V_*W*_R_ and *W*_V_*W*_I_, respectively. Dotted lines are theoretically simulated signals one could have in the absence of *W*_V_.

The experiment realizing the described direct and reciprocal scattering processes was performed at the High Resolution Dynamics Beamline P01 of the PETRA-III synchrotron source of the Deutsches Elektronen Synchrotron (DESY), which delivered *σ*-polarized beam in the form of periodic bunches with bunch period time *t*_B_ = 192 ns, one 44 ps long pulse per each bunch. The beam was scattered on a pair of ^57^Fe containing foils placed between a polarizer and an analyser, both having an extinction of 10^−8 ^26,27, and was subsequently detected by a Si avalanche photo diode (APD). The two foils were arranged according to the heterodyne setup^28^,^29^ (Figs 1 and 2d): One of them was a single-line stainless steel absorber, acting as a reference sample and mounted on a Mössbauer drive, and the other foil produced the polarization dependent scattering as a result of being magnetic. Each of the six high-frequency and narrow nuclear resonance signals in the magnetic foil gets superposed and produces beats with the corresponding nearby frequency resonance signal in the reference foil. These lower frequency beats are not only more easily detectable but, thanks to the heterodyne setup, also tuneable by the Doppler shift of energy caused by the velocity of the Mössbauer drive. After a pulse, resonances decay as time passes, and one detects counts – essentially, intensity – as a function of time as well as of the drive velocity. This intensity, *I(t, v*), gets multiplied, in the stroboscopic evaluation, by a window function *W(t*) and then integrated,


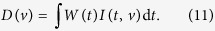


From the scattering matrix *S*, intensity can be calculated (see the Supplementary Information, Section S3) as


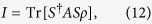


*ρ* denoting the density matrix for the incoming polarization and *A* the matrix expressing the effect of the analyser. Due to the periodic arrival of the bunches, *W(t*) can be expanded in Fourier series,


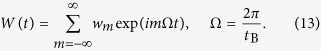


Correspondingly, using the Fourier transformed, frequency or energy, picture, *D(v*) can be expressed as 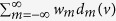, where the coefficients *d*_*m*_(*v*) are


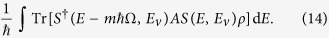


Therefore, while the *m* = 0 intensity is proportional to the squared magnitude of a certain scattering matrix element, stroboscopy brings in the existence of *m* ≠ 0 terms in which *S*^†^ and *S* appear at different energies, thus phase information also becomes accessible.

Seemingly, reciprocity violation in the phase wants to stay hidden even after these preparations because, at any *E*, either *S*^†^ or *S* is outside the narrow resonances that induce polarization dependent scattering, thus being approximately polarization independent, in other words, being proportional to the unit matrix. In this approximation, the two matrices belonging to the process *σ* → *π*,


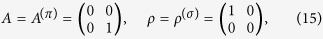


become neighbours under the trace and, since their product in either order is zero, the integrand is zero in this approximation (see Section S4 of the Supplementary Information). Nevertheless, this can be circumvented by observing that the sum of scattering intensities into any pair of orthogonal polarizations is independent of the choice of the pair of polarizations – namely, this sum is the intensity without any analyser. Hence, one has, for example,


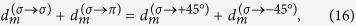


where *σ* → ±45° is scattering into polarization 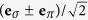 so we could extract 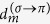 from measuring these three other quantities.

**Figure 3:**
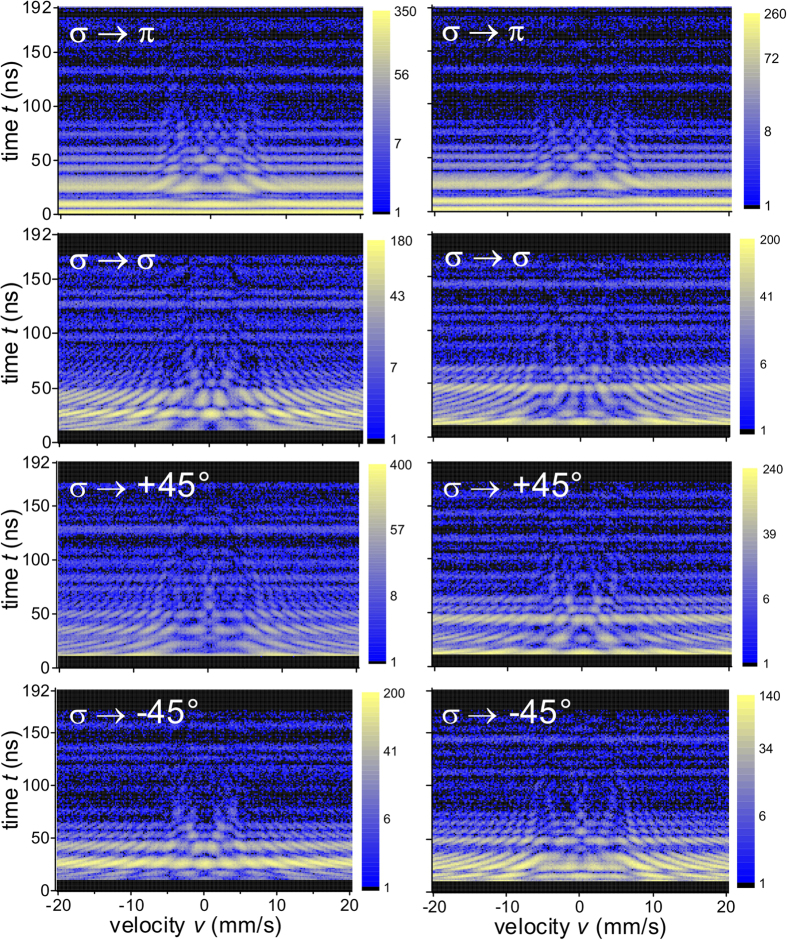
Direct (left column) and reciprocal (right column) scattering in the polarization dependent case. 2D intensity patterns of *σ* polarized synchrotron beam scattered into polarizations *π, σ*, +45° and −45°, respectively, when the second foil is a 6μm thick ferromagnetic ^57^Fe foil magnetized by permanent magnets in the direction of polar angles *ϑ* = 135°, *φ* = 90°. The two (direct and reciprocal) *σ* → *π* patterns are the same, indicating magnitude reciprocity (see also Section S2 of the Supplementary Information), while difference in the three other cases reports about reciprocity violation in the phase.

**Figure 4:**
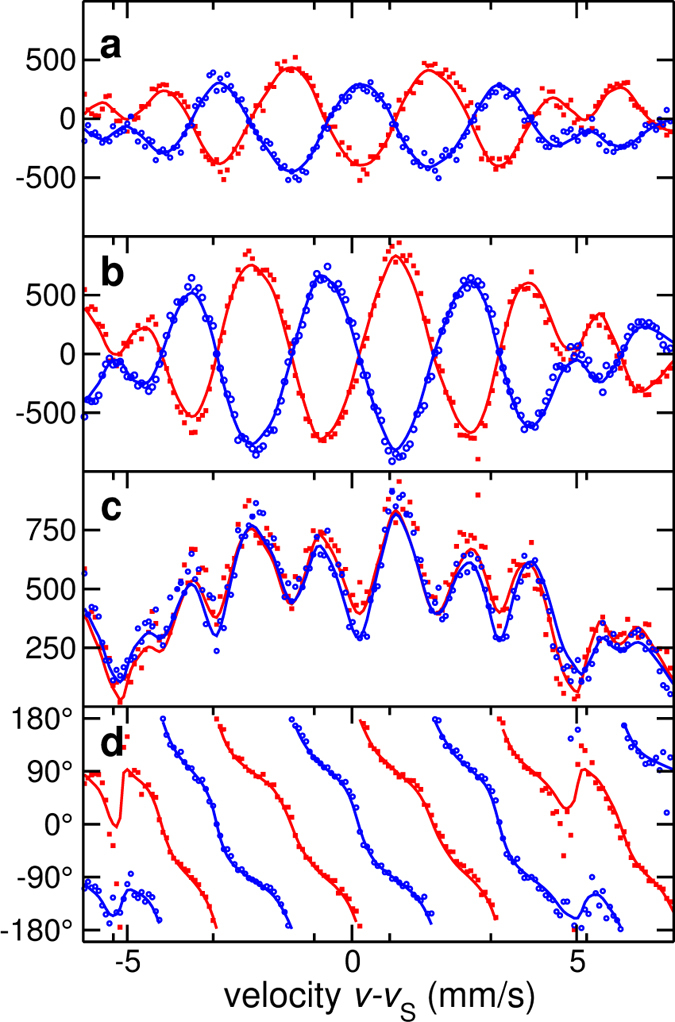
Real part (a), imaginary part (b), magnitude (c) and phase (d) of the complex scattering amplitude. The figure displays the range of the *m* = −1 order stroboscopic resonance; dots are experimental signals and continuous lines are theory simulations. The agreement between direct (red) and reciprocal (blue) data visible in (**c**) demonstrates magnitude reciprocity, while (**d**) shows maximal reciprocity violation in the phase (red and blue curves running with a 180° phase difference).

The experimental data and the results of the analysis are presented in Figs 2, 3 and 4. Figure 2 shows how the observed two-dimensional intensity patterns are processed via window functions to access the real and the imaginary part of the scattering amplitude. For calibration purposes, two stainless steel ^57^Fe foils have been measured (data displayed in Fig. 2). For all involved theoretical fittings and simulations, the computer framework EFFI^30^ has been used. Figure 3 shows the patterns when one of the samples is replaced by the polarization dependent scatterer (main experiment). Figure 4 displays the corresponding results of the evaluation of the patterns: the real part, imaginary part, magnitude and phase of the complex scattering amplitude. The predicted 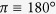 phase difference between direct and reciprocal data is apparent in Fig. 4d.

## Outlook

Both the present experimental arrangement and the one used for tuneable reciprocity violation^18^ have been devised utilizing the recent generalized reciprocity theorem^4^. This theoretical framework can similarly be used to contrive new measurement equipments and information technological devices that operate involving controlled spin/polarization dependent reciprocity behaviour. One example for this is based on the presence or absence of reciprocity in coupled two-mode systems, communicated in ref. 31.

## Additional Information

**How to cite this article:** Deák, L. *et al*. Realizing total reciprocity violation in the phase for photon scattering. *Sci. Rep.* **7**, 43114; doi: 10.1038/srep43114 (2017).

**Publisher's note:** Springer Nature remains neutral with regard to jurisdictional claims in published maps and institutional affiliations.

## Supplementary Material

Supplementary Information
