# Supplementary material for: Realizing total reciprocity violation in the phase for photon scattering
Source: Sci Rep. 2017 Feb 22;7:43114. doi: 10.1038/srep43114 (PMC5320471; doi:10.1038/srep43114)
Supplement: Supplementary Information [file srep43114-s1.pdf]

Realizing total reciprocity violation in the phase for photon scattering

László Deák[1*], László Bottyán[1], Tamás Fülöp[2], Dániel Géza Merkel[3,1], Dénes Lajos Nagy[1], Szilárd Sajti[1], Kai Sven Schulze[4,5], Hartmut Spiering[6], Ingo Uschmann[4,5], and Hans–Christian Wille[7]

[1]Wigner RCP, RMKI, P.O.B. 49, 1525 Budapest, Hungary

[2]Budapest University of Technology and Economics, 3 Műegyetem rkp., 1111 Budapest, Hungary

[3]European Synchrotron Radiation Facility, BP 220, 38043 Grenoble, France

[4]Helmholtz-Institut Jena, Fröbelstieg 3, 07743 Jena, Germany

[5]Friedrich-Schiller-Universität Jena, Max-Wien-Platz 1, 07743 Jena, Germany

[6]Johannes Gutenberg Universität Mainz, Staudinger Weg 9, 55099 Mainz, Germany

[7]Deutsches Elektronen-Synchrotron (PETRA III), Notkestrasse 85, 22607 Hamburg, Germany

[*]e-mail: deak.laszlo@wigner.mta.hu

Supplementary Information

Contents:

S1 Reciprocity in two polarization degrees of freedom

S2 Fixing the operator $U^{\mathrm{r}}U$ for nuclear resonant scattering

S3 The stroboscopic method for nuclear resonantly scattered gamma photons

S4 Solution of an orthogonality problem

S5 Standard and new stroboscopic window functions (with Supplementary Fig. S1)

S6 Experiments and data evaluation (with Supplementary Figs. S2–S6)

S1 Reciprocity in two polarization degrees of freedom

The generalized reciprocity theorem formulated in [4] for any elastic quantum or wave scattering problem becomes substantially simplified for forward and specular transmission/reflection on stratified media. Namely, in such cases the spatial propagation aspect is fixed and we only need to concentrate on what happens in polarization space. Here, we present details for the case of two spin/polarization components.

The scattered (transmitted or reflected) field Ψ_S can be expressed by the incident field Ψ_0 as

$$\Psi_S = S\Psi_0 \tag{S1}$$

with S being the 2×2 transmission (reflection) coefficient. Adopting the notation of Sturhahn *et al.* [24], transmissivity and reflectivity matrices will be commonly called *scattering matrices.* The scattered intensity, the scalar product $I = (\Psi_S, \Psi_S)$, depends on the polarization density matrix $\rho = \overline{\Psi_0 \otimes \Psi_0}$ of the incident plane wave [23],

$$I = \mathrm{Tr}\left[S^\dagger S\rho\right], \tag{S2}$$

where the overbar indicates averaging in time (dropped hereafter), $\dagger$ denotes the adjoint, $\otimes$ the tensorial product, and Tr stands for trace. Further scattering by an optical device, the so-called analyser described by a scattering matrix S_A, leads to the field $S_A S\Psi_0$, and the intensity becomes

$$I_A = \mathrm{Tr}\left[S^\dagger A S\rho\right], \tag{S3}$$

where the matrix $A = S_A^\dagger S_A$ represents the effect of the analyser.

In the ideal case when the incident beam is perfectly polarized in one of the directions of two orthonormal basis vectors $\mathbf{e}_1$, $\mathbf{e}_2$ in the polarization plane, and if the analyser also scatters perfectly into one of these directions, then the density matrix ρ and the analyser matrix A simplify to

$$\rho = \rho^{(j)} = \mathbf{e}_j \otimes \mathbf{e}_j \tag{S4}$$

and

$$A = A^{(i)} = \mathbf{e}_i \otimes \mathbf{e}_i \tag{S5}$$

so we have

$$I_A = |S_{ij}|^2 \tag{S6}$$

with the matrix element

$$S_{ij} = (\mathbf{e}_i, S\mathbf{e}_j). \tag{S7}$$

The four intensities $|S_{11}|^2$, $|S_{12}|^2$, $|S_{21}|^2$ and $|S_{22}|^2$ do not contain any information about the phases of the complex matrix elements. In Section S2 it is shown that the stroboscopic approach goes beyond (S3) and retains the phase information.

In the generalized reciprocity theorem [4], the central element is an antiunitary operator K. An antiunitary operator operates on wave functions in a product form

$$K = UJ, \tag{S8}$$

where J is complex conjugation and U is a unitary operator. The generalized reciprocity theorem asserts that, if the free Hamiltonian H_0 commutes with K and the potential V in the total Hamiltonian $H_0 + V$ satisfies $KVK^{-1} = V^\dagger$ then, stemming from the consequence $KG_E^\pm K^{-1} = G_E^{\pm\dagger}$ on the forward and backward Green's operators $G_E^\pm$, a property follows on the scattering matrix, which reads in our present simple case in the two-dimensional polarization space

$$KSK^{-1} = S^\dagger. \tag{S9}$$

With (S8), an equivalent form of this property is

$$S = US^\mathsf{T}U^{-1}, \tag{S10}$$

connecting S with its transpose S^T. From this latter form, it is apparent how the classic reciprocity condition $S = S^\mathsf{T}$ is covered as a special case, with U being the identity matrix.

Condition (S10) gives for the matrix elements (S7)

$$(\mathbf{e}_i, S\mathbf{e}_j) = \left(\mathbf{e}_j, S^\mathsf{T}\mathbf{e}_i\right) = \left(\mathbf{e}_j, U^{-1}SU\mathbf{e}_i\right) = \left(\mathbf{e}_j, U^\dagger SU\mathbf{e}_i\right) = (U\mathbf{e}_j, SU\mathbf{e}_i)\,. \tag{S11}$$

This result says that the scattering matrix element remains the same after interchange of source and detector (including $i, j \to j, i$) plus the transformation of polarizations by U.

When, because of practical reasons, the interchange of source and detector is realized via a 180° rotation of the sample, this means a transformation $S^\mathrm{r} = U^\mathrm{r}S(U^\mathrm{r})^{-1}$ on the scattering matrix S, where the unitary U^r is the representation of the rotation in polarization space [4]. Then (S11) can be further written as

$$(\mathbf{e}_i, S\mathbf{e}_j) = (U\mathbf{e}_j, SU\mathbf{e}_i) = (U^\mathrm{r}U\mathbf{e}_j, U^\mathrm{r}SU\mathbf{e}_i) = (U^\mathrm{r}U\mathbf{e}_j, S^\mathrm{r}U^\mathrm{r}U\mathbf{e}_i)\,. \tag{S12}$$

In this form, a scattering $\mathbf{e}_j \to \mathbf{e}_i$ is related to a one with polarizations $U^\mathrm{r}U\mathbf{e}_i \to U^\mathrm{r}U\mathbf{e}_j$.

Both mathematically and practically, the simplest cases are those when $U^\mathrm{r}U$ maps our basis vectors to basis vectors; in other words, when $U^\mathrm{r}U$ is a matrix in which only the two diagonal elements, or the two offdiagonal ones, are nonzero. Already these matrixes can introduce a complex phase factor on the basis vectors,

$$U^\mathrm{r}U\mathbf{e}_i = \lambda_{ij}\mathbf{e}_j \tag{S13}$$

with the coefficients λ_{ij} $(i, j = 1, 2)$ being complex numbers of magnitude 1. Correspondingly, (S12) gets shortened to

$$S_{ij} = \lambda_{jk}^*\lambda_{il}S_{kl}^\mathrm{r}, \tag{S14}$$

where * denotes complex conjugation. Polarization conserving scattering $(j = i,\ l = k)$ unavoidably leads to

$$\lambda_{jk}^*\lambda_{il} = 1, \qquad S_{ii} = S_{ll}^\mathrm{r} \tag{S15}$$

in both cases $(i = l,\ i \neq l)$ so no complex factor between the direct and reciprocal matrix elements can be achieved. In contrary, if polarization is changed during scattering $(i \neq j)$ then the product $\lambda_{jk}^*\lambda_{il}$ can be complex. Its phase is lost in experiments that are sensitive to intensities only, seeing only the magnitude $\left|\lambda_{jk}^*\lambda_{il}\right| = 1$. However, the novel stroboscopic method presented here is capable to capture the the phase, as shown in Section S3.

S2 Fixing the operator $U^{\mathrm{T}}U$ for nuclear resonant scattering

The polarization dependent part of the scattering potentials for the $M = -1, 0, 1$ Mössbauer transitions in case of ^{57}Fe are [4]

$$V_{-1} = \begin{pmatrix} 1-\sin^2\vartheta\sin^2\varphi & -i\cos\vartheta - \frac{\sin^2\vartheta\sin 2\varphi}{2} \\ i\cos\vartheta - \frac{\sin^2\vartheta\sin 2\varphi}{2} & 1-\cos^2\vartheta\sin^2\varphi \end{pmatrix}, \tag{S16}$$

$$V_{0} = \begin{pmatrix} 1-\sin^2\vartheta\cos^2\varphi & \frac{\sin^2\vartheta\sin 2\varphi}{2} \\ \frac{\sin^2\vartheta\sin 2\varphi}{2} & 1-\sin^2\vartheta\sin^2\varphi \end{pmatrix}, \tag{S17}$$

$$V_{+1} = \begin{pmatrix} 1-\sin^2\vartheta\sin^2\varphi & i\cos\vartheta - \frac{\sin^2\vartheta\sin 2\varphi}{2} \\ -i\cos\vartheta - \frac{\sin^2\vartheta\sin 2\varphi}{2} & 1-\cos^2\vartheta\sin^2\varphi \end{pmatrix} \tag{S18}$$

in the polarization basis distinguished by the synchrotron setup,

$$\mathbf{e}_1 = \mathbf{e}_\sigma, \qquad \mathbf{e}_2 = \mathbf{e}_\pi, \tag{S19}$$

both being orthogonal to the beam propagation direction $\mathbf{e}_3$, and the polar angles ϑ, φ understood with respect to these basis vectors. Since each of these transitions get excited during a scattering process, the generalized reciprocity condition $KVK^{-1} = V^\dagger$, also expressible as $V = UV^{\mathsf{T}}U^{-1}$, has to be valid with a U that relates each of the three potentials V_M to the corresponding transpose, $V_M = UV_M^{\mathsf{T}}U^{-1}$. As shown in [4], this is possible if and only if the three-vectors $\mathbf{b}_M$

$$\mathbf{b}_M = \frac{1}{2}\mathrm{Tr}(\boldsymbol{\sigma}V_M) \tag{S20}$$

of the Poincaré vector representation

$$V_M = b_0\sigma_0 + \mathbf{b}_M\boldsymbol{\sigma} \tag{S21}$$

are in a common plane, where $\boldsymbol{\sigma}$ comprises the components $\sigma_1, \sigma_2, \sigma_3$, and

$$\sigma_0 = \begin{pmatrix} 1 & 0 \\ 0 & 1 \end{pmatrix}, \quad \sigma_1 = \begin{pmatrix} 0 & 1 \\ 1 & 0 \end{pmatrix}, \quad \sigma_2 = \begin{pmatrix} 0 & -i \\ i & 0 \end{pmatrix}, \quad \sigma_3 = \begin{pmatrix} 1 & 0 \\ 0 & -1 \end{pmatrix}. \tag{S22}$$

Indeed, each of the matrices (S22) is self-transpose except for σ_2, which is anti-selftranspose. Therefore, transposition means for $\mathbf{b}_M$ that its second component changes sign and the other two components remain intact. In other words, transposition performs a reflection of $\mathbf{b}_M$ with respect to the σ_1–σ_3 plane. In parallel, the effect of U on $\mathbf{b}_M$ is a rotation by angle γ around real unit three-vector $\mathbf{n}$, both uniquely defined by the decomposition [25, p546]

$$U = e^{i\delta}\left(\cos\frac{\gamma}{2}\sigma_0 - i\sin\frac{\gamma}{2}\mathbf{n}\boldsymbol{\sigma}\right). \tag{S23}$$

$V_M = UV_M^{\mathsf{T}}U^{-1}$ means on $\mathbf{b}_M$ that its reflection with respect to the σ_1–σ_3 plane is compensated by an appropriate rotation. Now, to rotate back three vectors from their reflected is

geometrically possible if and only if the three vectors are in a common plane. For our V_Ms this is indeed the case, with

$$\mathbf{b}_{-1} = \begin{pmatrix} -\sin^2\vartheta \sin 2\varphi \\ -2\cos\vartheta \\ \sin^2\vartheta \cos 2\varphi \end{pmatrix}, \quad \mathbf{b}_0 = \begin{pmatrix} 2\sin^2\vartheta \sin 2\varphi \\ 0 \\ -2\sin^2\vartheta \cos 2\varphi \end{pmatrix}, \quad \mathbf{b}_{+1} = \begin{pmatrix} -\sin^2\vartheta \sin 2\varphi \\ 2\cos\vartheta \\ \sin^2\vartheta \cos 2\varphi \end{pmatrix}. \tag{S24}$$

The common rotation needed here is especially simple to give, since $\mathbf{b}_{-1}$ and $\mathbf{b}_{+1}$ are each other's reflected with respect to the σ_1–σ_3 plane. Namely, the 180° rotation around axis

$$\mathbf{n} = \begin{pmatrix} \sin 2\varphi \\ 0 \\ -\cos 2\varphi \end{pmatrix}, \tag{S25}$$

that is, around the direction of $\mathbf{b}_0$, maps $\mathbf{b}_{-1}$ to $\mathbf{b}_{+1}$ and vice versa, and keeps $\mathbf{b}_0$ invariant. In the language of U, with (S25), $\gamma = \pi$ and $\delta = \frac{3\pi}{2}$ (the value of the factor $e^{i\delta}$ actually being irrelevant for reciprocity as it drops out from any combination $U \cdots U^{-1}$), (S23) says

$$U = \begin{pmatrix} \cos 2\varphi & -\sin 2\varphi \\ -\sin 2\varphi & -\cos 2\varphi \end{pmatrix}. \tag{S26}$$

The wave vector $\mathbf{k}$ of the synchrotron beam pointing to the z direction, and the polarization basis vectors $\mathbf{e}_1$, $\mathbf{e}_2$ being the customary ones $\mathbf{e}_\sigma$ and $\mathbf{e}_\pi$, substituting the interchange of source and detector by a rotation of the sample means a 180° rotation around an arbitrary axis orthogonal to the z direction. If this axis includes angle α with $\mathbf{e}_\sigma$ in the $\mathbf{e}_\sigma$–$\mathbf{e}_\pi$ plane then $U^{\mathrm{r}}(\alpha)$ is calculated to be

$$U^{\mathrm{r}} = \begin{pmatrix} \cos 2\alpha & \sin 2\alpha \\ \sin 2\alpha & -\cos 2\alpha \end{pmatrix}. \tag{S27}$$

Then the product $U^{\mathrm{r}}U$ reads

$$U^{\mathrm{r}}U = \begin{pmatrix} \cos 2(\alpha+\varphi) & -\sin 2(\alpha+\varphi) \\ \sin 2(\alpha+\varphi) & \cos 2(\alpha+\varphi) \end{pmatrix}. \tag{S28}$$

As seen at the end of the previous section, we want $U^{\mathrm{r}}U$ to be offdiagonal, which can be achieved by $\cos 2(\alpha+\varphi) = 0$,

$$U^{\mathrm{r}}U = \pm \begin{pmatrix} 0 & 1 \\ -1 & 0 \end{pmatrix}. \tag{S29}$$

Then (S12) tells for the scattering matrix elements the relationships

$$S^{\mathrm{r}}_{\sigma\sigma} = S_{\pi\pi}, \qquad S^{\mathrm{r}}_{\sigma\pi} = -S_{\sigma\pi}, \tag{S30}$$

$$S^{\mathrm{r}}_{\pi\pi} = S_{\sigma\sigma}, \qquad S^{\mathrm{r}}_{\pi\sigma} = -S_{\pi\sigma}. \tag{S31}$$

Finally, the above conditions lead to

$$\alpha = 45^\circ - \varphi + k \cdot 90^\circ \tag{S32}$$

with any integer k. A convenient choice for our experiment is $\varphi = 90^\circ$ and $k = 1$, providing $\alpha = 45^\circ$. In order to avoid any zero trigonometrical factor in (S24), we have taken $\vartheta = 135^\circ$.

S3 The stroboscopic method for nuclear resonantly scattered gamma photons

The setup of a heterodyne/stroboscopic nuclear resonance scattering (NRS) of synchrotron radiation (SR) experiment includes two scatterers in forward scattering geometry, one being the investigated specimen and the other a reference sample mounted on a Mössbauer drive [28, 29, 21, 20]. In our experiment, the specimen is a ferromagnetic α–^{57}Fe foil with six Mössbauer resonances $E_1, \ldots, E_6$, and the reference sample is a stainless steel foil with a single line at E_0, which is shifted to $E_0 + E_v$ via the Doppler effect. The combined scatterer behaves differently for different E_v so the combined scattering matrix depends on both the incoming energy E and the shifted energy E_v, $S = S\left(E, E_v\right)$.

An NRS experiment measures the delayed intensity after simultaneous excitation of the nuclei of the scatterer by the synchrotron pulse. With t denoting the time after the synchrotron excitation, the scattering matrix $S\left(t, E_v\right)$ is the Fourier transform of the energy domain scattering matrix $S\left(E, E_v\right)$. The function studied in heterodyne spectroscopy is the delayed photon rate $D\left(E_v\right)$, which, similarly to a Mössbauer spectrum, depends on the Doppler velocity v. D is defined as the time integral

$$D\left(E_v\right) = \int_{-\infty}^{\infty} \mathrm{d}t W\left(t\right) I(t, E_v) \tag{S33}$$

with the delayed intensity

$$I\left(t, E_v\right) = \mathrm{Tr}\left[S^{\dagger}\left(t, E_v\right) A S\left(t, E_v\right) \rho\right] \tag{S34}$$

[cf. (S3)] and a time window function $W\left(t\right)$ that is either realized within the experiment or introduced during processing the measured data $I\left(t, E_v\right)$.

It is the time window function that extracts various answers from the two-dimensional area of measured intensities [21, 22]. Simple boxcar functions,

$$W(t) = \begin{cases} 1 & \text{when} \quad t_1 + n t_\mathrm{B} < t < t_2 + n t_\mathrm{B} \quad \text{(for all integers } n\text{)}, \\ \\ 0 & \text{otherwise,} \end{cases} \tag{S35}$$

with t_B being the time interval between the synchrotron bunches, have been applied in order to obtain spectra similar to Mössbauer absorption spectra.

Due to the bunch periodicity, the time window function can be expanded in a Fourier series [21, 20],

$$W\left(t\right) = \sum_{m=-\infty}^{\infty} w_m \exp\left(im\Omega t\right), \qquad \Omega = \frac{2\pi}{t_\mathrm{B}} \tag{S36}$$

so the delayed count rate, combining (S34)–(S36), can be written as an infinite sum,

$$D\left(E_v\right) = \sum_{m=-\infty}^{\infty} w_m d_m\left(E_v\right) \tag{S37}$$

with

$$d_m\left(E_v\right) = \frac{1}{\hbar}\int \mathrm{d}E\mathrm{Tr}\left\{\left[S\left(E-m\varepsilon,E_v\right)-S_\infty\right]^\dagger A\left[S\left(E,E_v\right)-S_\infty\right]\rho\right\}, \tag{S38}$$

where

$$\varepsilon = \hbar\Omega = \hbar\frac{2\pi}{t_\mathrm{B}} \tag{S39}$$

and S_∞ is the scattering matrix far from resonances; S_∞ is proportional to the unit matrix since it describes polarization independent scattering. It is subtracted because it causes a prompt Dirac delta-like contribution at $t = 0$ and does not contribute to the *delayed* count rate [22] and we wish to Fourier transform only the delayed intensity.

As the time window $W(t)$ and the intensity $D(E_v)$ are real functions, $w_m = w^*_{-m}$ and $d_m = d^*_{-m}$ follow, and thus (S37) is expressible as

$$D\left(E_v\right) = w_0 d_0 + \sum_{m=1}^{\infty} 2\,\mathrm{Re}\left[w_m d_m(E_v)\right]. \tag{S40}$$

The result (S37)–(S39) is a direct generalization of the intensity formula (S34) to the heterodyne/stroboscopic NRS of SR for any observed channel in the applied experimental geometry. This expression has already been derived for the case of forward scattering [21, 20]. The $m = 0$ term has been called the 'heterodyne spectrum' [28, 21] and the $m \geq 1$ terms 'stroboscopic resonances' [21] of order m. The stroboscopic resonances are not restricted to the forward scattering case but also appear in other experimental geometries including grazing incidence scattering or Bragg diffractions.

The $m = 0$ term does not contain phase information so the first task is to understand the behavior of the $d_{m\geq 1}$ terms. Then, since (S40) represents a system of linear equations for the d_ms with different coefficient sets w_m realizable with different time window functions, the subsequent question is the choice of appropriate time window functions, studied in Sect. S5.

The properties of $d_{m\geq 1}$ stem from those of S. The scattering matrix S describes a combined scatterer. In the α–^{57}Fe scatterer, there are six resonance lines at E_p, $p = 1, \ldots, 6$, with finite width and Lorentzian shape; the six resonances have insignificant overlap. In parallel, in the reference scatterer, there is one resonance of similar width at energy $E_0 + E_v$. $S(E, E_v)$ differs considerably from S_∞ only if the incoming energy E is in the vicinity of one of these energies E_p, $E_0 + E_v$. Scattering related to the E_ps is polarization dependent while the resonance $E_0 + E_v$ provides polarization independent scattering. Also notably, we can assume – and will take advantage of – that the lines E_p are closer to each other than the energy shift ε,

$$E_6 - E_1 < \varepsilon. \tag{S41}$$

In order to get significant contribution to the integral (S38) over energy E, both brackets $[\cdots]$ in its integrand have to be different from zero. As we have seen, this requires, on the side of the right bracket,

$$E \simeq E_p \quad \text{or} \quad E \simeq E_0 + E_v. \tag{S42}$$

The left bracket induces an analogous condition on the shifted energy argument:

$$E - m\varepsilon \simeq E_q \quad \text{or} \quad E - m\varepsilon \simeq E_0 + E_v. \tag{S43}$$

To see when these both can hold, we need to investigate the following four pairings of requirements:

$$
\begin{array}{rcccll}
E_q + m\varepsilon & \simeq & E & \simeq & E_p : & \text{impossible as } |E_q - E_p| < \varepsilon, \\
E_q + m\varepsilon & \simeq & E & \simeq & E_0 + E_v : & \text{possible,} \\
E_0 + E_v + m\varepsilon & \simeq & E & \simeq & E_p : & \text{possible,} \\
E_0 + E_v + m\varepsilon & \simeq & E & \simeq & E_0 + E_v : & \text{impossible as } \varepsilon > \text{line width of } E_0 + E_v.
\end{array} \tag{S44}
$$

The two possible cases can be summarized for E_v in the form that $d_m(E_v)$ is non-negligible only if, with some p,

$$E_v \simeq E_p - E_0 + m\varepsilon \qquad \text{or} \tag{S45}$$

$$E_v \simeq E_p - E_0 - m\varepsilon. \tag{S46}$$

On the other side, we can also read off from (S44) that, even for such a E_v fixed, the integral (S38) over E collects contribution only from a single narrow domain, the overlap of

$$E \simeq E_p + m\varepsilon \quad \text{and} \quad E \simeq E_0 + E_v \qquad \text{or of} \tag{S47}$$

$$E \simeq E_0 + E_v + m\varepsilon \quad \text{and} \quad E \simeq E_p, \tag{S48}$$

respectively. With the rearranging

$$E \simeq E_0 + E_v + m\varepsilon = E_0 + (E_v - n\varepsilon) + (m+n)\varepsilon \tag{S49}$$

we can recognize that

$$d_m(E_v) = d_{m+n}(E_v - n\varepsilon) \tag{S50}$$

holds for (S46), and, via an analogous argument,

$$d_m(E_v) = d_{m-n}(E_v + n\varepsilon) \tag{S51}$$

is fulfilled for the case (S45). This means that all the higher order stroboscopic resonances provide the same physical information about the scatterer as d_1. This explains why, occasionally, a d_m itself is also called scattering amplitude. It also enables us to focus on d_1 only in subsequent sections.

The forthcoming considerations make use of another property of $d_m(E_v)$ as well. Namely, (S44), we have seen that, whenever both brackets $[\cdots]$ in (S38) are non-negligible, only one of them can be related to a polarization dependent resonance. Hence, the other one – being proportional to the unit matrix – can be moved before the trace. This yields

$$
d_m(E_v) \approx \begin{cases}
\frac{1}{\hbar}\int \mathrm{d}E \left[S(E,E_v) - S_\infty\right]_0 \mathrm{Tr}\left\{\left[S(E-m\varepsilon,E_v) - S_\infty\right]^\dagger A\rho\right\} \\
\qquad\qquad\qquad\qquad\qquad\qquad \text{if } \; E_v \simeq E_p - E_0 + m\varepsilon, \\
\frac{1}{\hbar}\int \mathrm{d}E \left[S(E-m\varepsilon,E_v) - S_\infty\right]_0^* \mathrm{Tr}\left\{\left[S(E,E_v) - S_\infty\right]\rho A\right\} \\
\qquad\qquad\qquad\qquad\qquad\qquad \text{if } \; E_v \simeq E_p - E_0 - m\varepsilon,
\end{cases} \tag{S52}
$$

where the factors before the traces are the scalars that multiply the unit matrix [in other words, the zeroth Poincaré coefficients in the (S21)-type decomposition].

S4 Solution of an orthogonality problem

In the polarization basis (S19), the polarization density (S4) of the σ-polarized incident synchrotron radiation reads

$$\rho^{(\sigma)} = \begin{pmatrix} 1 & 0 \\ 0 & 0 \end{pmatrix}, \tag{S53}$$

and scattering into polarization directions $\mathbf{e}_\sigma$, $\mathbf{e}_\pi$, $(\mathbf{e}_\sigma + \mathbf{e}_\pi)/\sqrt{2}$ and $(\mathbf{e}_\sigma - \mathbf{e}_\pi)/\sqrt{2}$ are described by the analysers

$$A^{(\sigma)} = \begin{pmatrix} 1 & 0 \\ 0 & 0 \end{pmatrix}, \quad A^{(\pi)} = \begin{pmatrix} 0 & 0 \\ 0 & 1 \end{pmatrix}, \quad A^{(+45^\circ)} = \frac{1}{2}\begin{pmatrix} 1 & 1 \\ 1 & 1 \end{pmatrix}, \quad A^{(-45^\circ)} = \frac{1}{2}\begin{pmatrix} 1 & -1 \\ -1 & 1 \end{pmatrix}, \tag{S54}$$

respectively [see (S5)].

The minus sign in $S^{\mathrm{r}}{}_{\pi\sigma} = -S_{\pi\sigma}$ of (S31) offers a nice possibility to demonstrate maximal reciprocity violation in phase. Seemingly, one only needs to measure the scattering $\sigma \to \pi$ in the direct and the reciprocal setting and analyse the data via some appropriate window functions. However, the products $A^{(\pi)}\rho^{(\sigma)}$, $\rho^{(\sigma)}A^{(\pi)}$ appearing in the resonant approximation (S52) are zero so $\sigma \to \pi$ scattering data does not really provide information about the matrix element $S_{\pi\sigma}$. Nevertheless, this orthogonality-related inconvenience can be circumvented. Namely, one can have access to $S_{\pi\sigma}$ via other scattering processes such as $\sigma \to \sigma$, $\sigma \to +45^\circ$ and $\sigma \to -45^\circ$.

The first ingredient for this is the property

$$d_m^{(\sigma\to\sigma)} + d_m^{(\sigma\to\pi)} = d_m^{(\sigma\to+45^\circ)} + d_m^{(\sigma\to-45^\circ)}, \tag{S55}$$

a consequence of

$$A^{(\sigma)} + A^{(\pi)} = A^{(+45^\circ)} + A^{(-45^\circ)} = \begin{pmatrix} 1 & 0 \\ 0 & 1 \end{pmatrix}, \tag{S56}$$

in view of the definition of the d_ms, (S38). In fact, the sum of intensities scattering into any pair of orthogonal polarizations is nothing but the intensity of scattering without any analyser.

In parallel to this exact relationship, we have the approximate ones

$$d_m^{(\sigma\to\sigma)}(E_v) \approx \tfrac{1}{\hbar}\int \mathrm{d}E\, [S(E-m\varepsilon, E_v) - S_\infty]_0^* \, [S_{\sigma\sigma}(E,E_v) - S_\infty], \tag{S57}$$

$$d_m^{(\sigma\to\pi)}(E_v) \approx 0, \tag{S58}$$

$$d_m^{(\sigma\to+45^\circ)}(E_v) \approx \tfrac{1}{\hbar}\int \mathrm{d}E\, [S(E-m\varepsilon, E_v) - S_\infty]_0^* \left[\tfrac{1}{2}S_{\sigma\sigma}(E,E_v) - \tfrac{1}{2}S_\infty + \tfrac{1}{2}S_{\pi\sigma}(E,E_v)\right], \tag{S59}$$

$$d_m^{(\sigma\to-45^\circ)}(E_v) \approx \tfrac{1}{\hbar}\int \mathrm{d}E\, [S(E-m\varepsilon, E_v) - S_\infty]_0^* \left[\tfrac{1}{2}S_{\sigma\sigma}(E,E_v) - \tfrac{1}{2}S_\infty - \tfrac{1}{2}S_{\pi\sigma}(E,E_v)\right], \tag{S60}$$

according to (S52) with our choice (S46). These approximate formulae are consistent with (S55), as can be readily seen. Moreover, how $S_{\pi\sigma}(E, E_v)$ cancels out from the *sum* of the

right hand sides of (S59) and (S60) gives the idea to form the *difference* of these two right hand sides in order to access $S_{\pi\sigma}(E, E_v)$. More closely, we can define

$$d_m^{\text{combined}}\left(E_v\right) = d_m^{(\sigma\to+45^\circ)}(E_v) - d_m^{(\sigma\to-45^\circ)}(E_v) + d_m^{(\sigma\to\pi)}(E_v), \tag{S61}$$

which corresponds to substituting

$$A^{\text{combined}} = A^{(+45^\circ)} - A^{(-45^\circ)} + A^{(\pi)} = \begin{pmatrix} 0 & 1 \\ 1 & 1 \end{pmatrix} \tag{S62}$$

into (S38) in the place of A.

To see how $d_m^{\text{combined}}\left(E_v\right)$ behaves with respect to reciprocity, let us introduce the shorthands

$$T\left(E, E_v\right) = S\left(E, E_v\right) - S_\infty, \qquad \bar{T}\left(E, E_v\right) = S\left(E - m\varepsilon, E_v\right) - S_\infty, \tag{S63}$$

with which $d_m^{\text{combined}}\left(E_v\right)$ can be expressed as

$$d_m^{\text{combined}}\left(E_v\right) = \frac{1}{\hbar}\int \mathrm{d}E\left[\bar{T}^*_{\sigma\sigma}T_{\pi\sigma} + \bar{T}^*_{\pi\sigma}T_{\sigma\sigma} + \bar{T}^*_{\pi\sigma}T_{\pi\sigma}\right]\left(E, E_v\right). \tag{S64}$$

Here, the third term in the bracket corresponds to the small $d_m^{(\sigma\to\pi)}(E_v)$ contribution. In the dominant first two terms, the offdiagonal factors change sign under reciprocity: (S31) yields

$$T^{\mathrm{r}}_{\pi\sigma} = -T_{\pi\sigma}, \qquad \left(\bar{T}^*_{\pi\sigma}\right)^{\mathrm{r}} = -\bar{T}^*_{\pi\sigma}. \tag{S65}$$

In the energy integral (S64), these offdiagonal factors are relevant at the polarization dependent resonances, as explained in the previous section. The diagonal factors multiplying them have resonance stemming from the polarization independent other sample, therefore, at those energies, T is essentially proportional to the unit matrix,

$$T_{\sigma\sigma} \approx T_{\pi\pi} = \left(T_{\sigma\sigma}\right)^{\mathrm{r}}, \qquad \bar{T}^*_{\sigma\sigma} \approx \bar{T}^*_{\pi\pi} = \left(\bar{T}^*_{\sigma\sigma}\right)^{\mathrm{r}} \tag{S66}$$

bearing in mind (S30), and hence,

$$\left(\bar{T}^*_{\sigma\sigma}T_{\pi\sigma}\right)^{\mathrm{r}} \approx -\bar{T}^*_{\sigma\sigma}T_{\pi\sigma}, \qquad \left(\bar{T}^*_{\pi\sigma}T_{\sigma\sigma}\right)^{\mathrm{r}} \approx -\bar{T}^*_{\pi\sigma}T_{\sigma\sigma}, \tag{S67}$$

and, altogether,

$$\left(d_m^{\text{combined}}\right)^{\mathrm{r}} \approx -d_m^{\text{combined}} \tag{S68}$$

so reciprocity violation is nicely visible.

In practice, one does not measure $d_m^{(\sigma\to\pi)}$, $d_m^{(\sigma\to+45^\circ)}$ and $d_m^{(\sigma\to-45^\circ)}$ – in other words, $D^{(\sigma\to\pi)}$, $D^{(\sigma\to+45^\circ)}$ and $D^{(\sigma\to-45^\circ)}$ – but detector counts $C^{(\sigma\to\pi)}$, $C^{(\sigma\to+45^\circ)}$ and $C^{(\sigma\to-45^\circ)}$ collected during a certain time period, and the different scattering processes run for different time periods and with slightly varying synchrotron intensities. Hence, for combining data about these processes, one needs to introduce a relative normalization of the three different data sets. To this end, we have measured the four quantities $C^{(\sigma\to\sigma)}$, $C^{(\sigma\to\pi)}$, $C^{(\sigma\to+45^\circ)}$ and $C^{(\sigma\to-45^\circ)}$ as functions of E_v, fitted three relative normalizations $\lambda^{(\sigma\to\pi)}$, $\lambda^{(\sigma\to+45^\circ)}$ and $\lambda^{(\sigma\to-45^\circ)}$ with which the relationship among these functions that corresponds to (S55), i.e.,

$$C^{(\sigma\to\sigma)}(E_v) + \lambda^{(\sigma\to\pi)}C^{(\sigma\to\pi)}(E_v) = \lambda^{(\sigma\to+45^\circ)}C^{(\sigma\to+45^\circ)}(E_v) + \lambda^{(\sigma\to-45^\circ)}C^{(\sigma\to-45^\circ)}(E_v), \tag{S69}$$

holds the best, and then used these relative normalizations to reconstruct (S61), in other words, $D^{\text{combined}}(E_v)$.

S5 Standard and new stroboscopic window functions

The classic window function used for stroboscopy [21] is the already mentioned single boxcar-type one,

$$W_0(t) = \begin{cases} 1 & \text{when} \quad t_1 + nt_\mathrm{B} < t < t_2 + nt_\mathrm{B} \quad \text{(for all integers } n\text{)}, \\ \\ 0 & \text{otherwise.} \end{cases} \tag{S70}$$

The corresponding discrete Fourier components, cf. (S36), are

$$(w_0)_m = \exp\left(-2i\pi m\eta\right) \frac{\sin\left(m\pi F\right)}{m\pi}, \tag{S71}$$

where $\eta = (t_2 + t_1)/\left(2t_\mathrm{B}\right)$ characterizes the position of the time window and $F = (t_2 - t_1)/t_\mathrm{B}$ its width, both in units of t_B. For $\eta = 1/2$, $F = 1/2$, the Fourier components are real, and are nonzero for odd ms only [21]. As a consequence, the stroboscopic intensity $D_0\left(E_v\right)$, see (S40), depends only on the real part of d_m. Here, we wish to obtain information about the imaginary part, too. To this end, let us shift the classic windows by $\pm\Delta\eta$ (while keeping $F = 1/2$):

$$W_0^+(t) = W_0\left(t + t_\mathrm{B}\Delta\eta\right), \qquad W_0^-(t) = W_0\left(t - t_\mathrm{B}\Delta\eta\right), \tag{S72}$$

which involves two new stroboscopic intensities in addition to the classic one:

$$D_0 = (w_0)_0 d_0 - 2\sum_{m=1}^{\infty} \left|(w_0)_m\right| \left|d_m\right| \cos\varphi_m, \tag{S73}$$

$$D_0^\pm = (w_0)_0 d_0 - 2\sum_{m=1}^{\infty} \left|(w_0)_m\right| \left|d_m\right| \cos\left(\mp 2\pi m\Delta\eta + \varphi_m\right), \tag{S74}$$

where φ_m is the phase of the complex number d_m, written out explicitly to help to find – via trigonometric identities – that the real and imaginary parts can be expressed separately:

$$\frac{1}{2}\left(D_0^+ + D_0^-\right) - D_0 = 4\sum_{m=1}^{\infty} \left|(w_0)_m\right| \sin^2\left(\pi m\Delta\eta\right) \mathrm{Re}(d_m), \tag{S75}$$

$$\frac{1}{2}\left(D_0^+ - D_0^-\right) = 2\sum_{m=1}^{\infty} \left|(w_0)_m\right| \sin\left(2\pi m\Delta\eta\right) \mathrm{Im}(d_m). \tag{S76}$$

Here, the left-hand sides can be extracted from experimental data, and $\Delta\eta$ can be chosen freely.

Note that the inconvenient 0th order stroboscopic resonance d_0 is missing from (S75)–(S76), and (thanks to $F = 1/2$) the odd stroboscopic resonances are actually also absent. In parallel, according to (S71), $(w_0)_m$ decreases with increasing m as $\left|(w_0)_m\right| \sim 1/m$. All these improve the possibility to extract the quantities $\mathrm{Re}(d_m)$, $\mathrm{Im}(d_m)$ themselves, by reducing the overlap of distinct resonances. We remark that, should it be needed, the system of equations (S75)–(S76) can be extended to any number using various different $\Delta\eta$s. Also notably,

according to the properties (S50)–(S51), already the coefficient d_1 carries all scattering information.

Assuming that the fifth and higher orders are negligible in (S75)–(S76), now let us see how the choice of $\Delta\eta$ can be optimized. Our aim is to extract d_1 so we can try to find a $\Delta\eta$ with which the coefficients of d_3 in (S75)–(S76) are zero:

$$\sin^2(3\pi\Delta\eta) = 0, \tag{S77}$$

$$\sin(6\pi\Delta\eta) = 0. \tag{S78}$$

The smallest $\Delta\eta$ ensuring both of these is $\Delta\eta = 1/3$. Unfortunately, this case cannot be realized, because then time window $[t_1, t_2]$ extends by $(1/12)t_{\mathrm{B}}$ beyond the synchrotron bunch window at one of its endpoints. However, a still satisfactory solution is to take $\Delta\eta = 1/6$, which makes the coefficient of the imaginary part of d_3 zero. Then we have three unknowns, $\mathrm{Re}(d_1)$, $\mathrm{Im}(d_1)$ and $\mathrm{Re}(d_3)$, in the three equations expressing D_0^+, D_0^- and D_0^0, enabling us to express the unknowns. Hence, d_1 is determined:

$$\mathrm{Re}(d_1) = \pi\left[\frac{1}{2}\left(D_0^+ + D_0^-\right) - D_0^0\right], \tag{S79}$$

$$\mathrm{Im}(d_1) = \frac{\pi}{2\sqrt{3}}\left(D_0^+ - D_0^-\right). \tag{S80}$$

Notably, (S75)–(S76) correspond to the two new window functions,

$$W_{\mathrm{R}} = \frac{1}{2}(W_+ + W_-) - W_0, \tag{S81}$$

$$W_{\mathrm{I}} = \frac{1}{2}(W_+ - W_-), \tag{S82}$$

with corresponding Fourier components

$$(w_{\mathrm{R}})_m = (w_0)_m\left[\cos\left(\frac{m\pi}{3}\right) - 1\right], \tag{S83}$$

$$(w_{\mathrm{I}})_m = -i(w_0)_m \sin\left(\frac{m\pi}{3}\right) \tag{S84}$$

for $\Delta\eta = 1/6$.

So far it has been assumed that the fifth and higher resonances are not overlapping with the first one. Now, the separation of stroboscopic resonances of increasing order by $\varepsilon = \hbar\frac{2\pi}{t_{\mathrm{B}}}$ is completely determined by the synchrotron bunch time t_{B}. Since ε should be larger than the hyperfine splitting of $\approx 10\,\mathrm{mm/s}$ in case of ferromagnetic α–Fe, the required bunch time should be $t_{\mathrm{B}} < 8\,\mathrm{ns}$.

In the experimental setup available in our case, the synchrotron source operated with $t_{\mathrm{B}} = 192$ ns. We have circumvented this inconvenience by a further enhancement to the window functions: we have changed their period of time from t_{B} to t_{B}/L – in other words, we have applied a train of L boxcars between any two synchrotron pulses. With the choice $L = 32$, the energy distance $L\epsilon = \hbar\frac{2\pi L}{t_{\mathrm{B}}}$ was already large enough to reduce the overlap

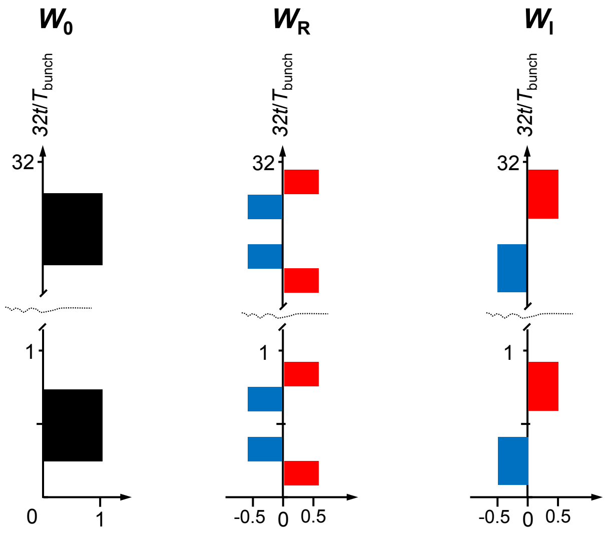

Figure S1: Time windows used for the stroboscopic evaluation. Classic (W_0), real part (W_R) and imaginary part (W_I) evaluation.

satisfactorily. The corresponding new Fourier components are $\left({w_0}^{(L)}\right)_{mL} = (w_0)_m$, and are zero for other integer indices.

Experimental conditions (mainly: detector dead times) add some veto (disable a certain time interval for data collecting within each bunch period), the effect of which can be expressed via a veto window function W_V. This multiplies the already chosen window functions, which means in the language of the Fourier components the convolution

$$\left(w_0^{\mathrm{effective}}\right)_m = \sum_{n=-\infty}^{\infty} \left({w_0}^{(L)}\right)_n (w_\mathrm{V})_{m-n} \,. \tag{S85}$$

In our choice, the veto window was positioned symmetrically ($\eta = 1/2$) and with a width parameter F_V [cf. (S71)]. The presence of such a veto window function has some slight consequences but the reciprocity violating minus sign seen in (S68) remains untouched.

S6 Experiments and data evaluation

The experiment was carried out in four steps. The first necessary preliminary experiment was the calibration of the reference sample, when its effective thickness is determined and the uniformness of the width is checked. The calibration has been made on two identical ^{57}Fe stainless steel (SS) foils, samples A and B cut from a larger uniform foil. Time resolved nuclear resonance forward scattering experiment performed on samples A and B are shown in Fig. S2. Since there is no hyperfine splitting at the Fe sites, in SS only dynamical beats appear, which are at the same position for both foils, proving their thicknesses to be equal. The electronic veto was set to 0–13 ns and 172–192 ns. Since there appear some rapid

oscillations up to 25 ns, typically caused by the dead time of the APD detector, this time region has been discarded by the veto window function W_V of Fig. 1 of the main text. The data has been fitted (see Fig. S2) using the nuclear resonance scattering simulation program EFFI (**E**nvironment **F**or **FI**tting) [30]. An outcome of the fit is the effective thickness $\alpha = \sigma_0 f N d$, where $\sigma_0 = 2.557 \cdot 10^{-18}$ cm^2 is the resonant cross-section, $f = 0.8$ the Lamb–Mössbauer factor, and Nd the number of resonant nuclei per area. The fitted values $\alpha_A = 19.97 \pm 0.04$ and $\alpha_B = 20.04 \pm 0.04$ are the same within the statistical errors, justifying that the thickness is uniform and that the two foils are of the same width.

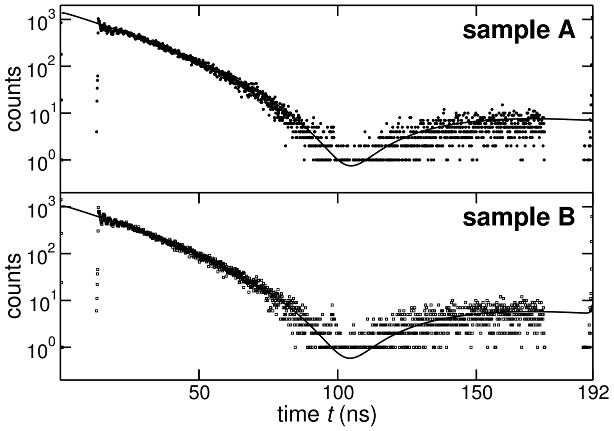

Figure S2: Calibrating the two ^{57}Fe stainless steel foils, sample A and sample B. Nuclear resonance forward scattering measurement data and simulation fits (solid lines).

The second calibrating and testing step was a heterodyne 2D measurement with sample A mounted on the Mössbauer drive and sample B being at rest – see Fig. 1 of the main text. The stroboscopic spectra by standard, real and imaginary part evaluation of the delayed photon rate $D(E_v)$ (shown in Figs. 1a, 1b and 1c, respectively) have been obtained using the window functions being based on $L = 32$ boxcars, from which train the veto window function excluded 4 boxcars. The result of the simultaneous fit of the three spectra – done with EFFI – is the maximal velocity $v_{max} = 20.38$ mm/s of the drive that operated in triangular constant acceleration mode. In addition, the fit shows that the nonlinearity of the velocity to channel conversion was negligible.

Third, the α–^{57}Fe foil sample providing polarization dependent scattering needed to be checked. To this end, nuclear resonance forward scattering experiments have been carried out on the α–^{57}Fe sample, measuring $\sigma \to \pi$, $\sigma \to \sigma$, $\sigma \to +45°$ and $\sigma \to -45°$ scattering in direct as well as reciprocal arrangement. Specifically, the synchrotron provided σ-polarized beam, and time diagrams were measured for the four analyser orientations π, σ, $+45°$ and $-45°$, each in direct and reciprocal arrangement both, which were realized as two different orientations of the magnetized foil, the direction of the magnetizing field of 0.05 T being at polar angles $\vartheta = 135°$, $\varphi = 180°$ in the direct case and at $\vartheta = 45°$, $\varphi = 270°$ in the reciprocal one.

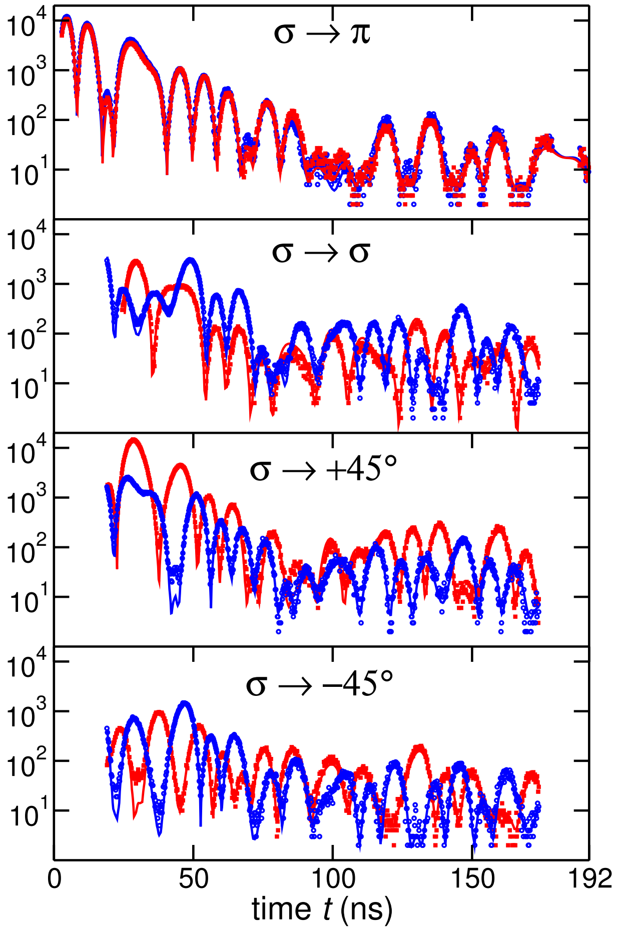

Figure S3: Direct (red) and reciprocal (blue) scattering processes on the α–^{57}Fe foil. Experimental results for the nuclear resonance forward scattering processes $\sigma \to \pi$, $\sigma \to \sigma$, $\sigma \to +45°$ and $\sigma \to -45°$. Solid lines show the simultaneous fits. Note that, for the $\sigma \to \pi$ case, which suppresses the prompt by 10^{-8}, there is no electronic veto (apart from a veto between 175 ns and 185 ns because of technical reasons).

The obtained eight time spectra can be seen in Fig. S3. The agreement between direct and reciprocal outcomes in $\sigma \to \pi$ scattering demonstrates that magnitude reciprocity, i.e., equality of the absolute values of scattering amplitudes, is present. (This is the level one can reach without phase sensitive procedures like the one we have accomplished in the fourth, main, experimental step.)

The eight spectra were simultaneously fitted (again using the simulation program EFFI). The magnetizing field of 0.05 T parallel to the plane of the foil was not large enough to uniformly magnetize the foil. This circumstance caused an angle distribution of the direction of the induced hyperfine field in the plane of the foil, symmetric around the direction of the external field. As a simple yet satisfactory approach, we have found that the angle

distribution can be substituted by a main field parallel to the external field with a certain weight, accompanied by two 'satellite' field vectors with equal weights and at angles $+\zeta$ resp. $-\zeta$ around the main direction. More closely, with $\zeta = 26.6°$, satellite weights $(15.4 \pm 2.0)\,\%$ and main component weight $(69.2 \pm 2.0)\,\%$, the mean squares value $\chi^2 = 5.2$ of the fit did not deteriorate remarkably. The three components were (32.84 ± 0.002) mT in magnitude.

While a much stronger external magnetic field could provide uniform magnetization of the foil, the presence of satellite components does not change the conclusions regarding reciprocity since all hyperfine field components still lie within a single plane, as explained in Section S2.

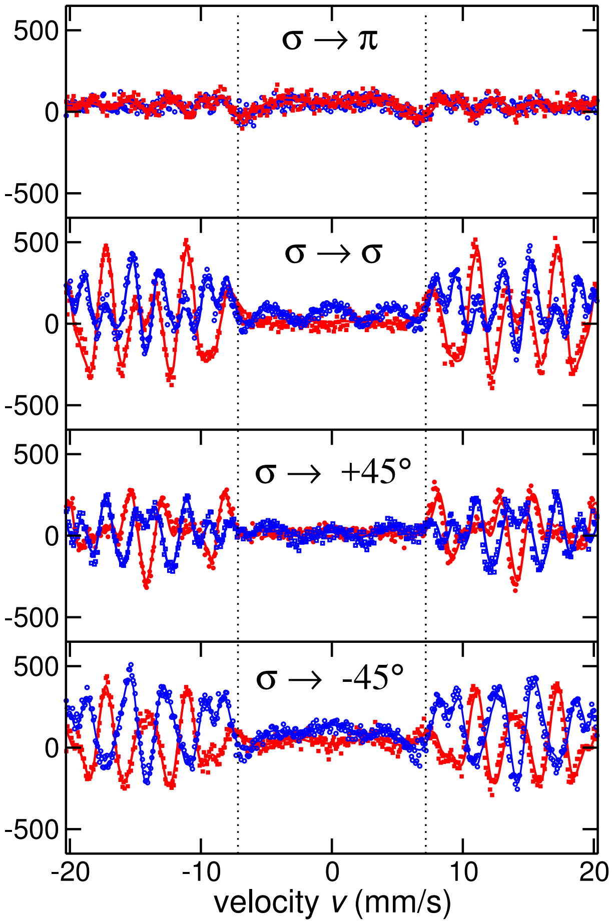

Figure S4: Real part stroboscopic evaluation of direct (red) and reciprocal (blue) scatterings on the α–^{57}Fe foil. Solid lines denote the simultaneous fits on the four nuclear resonance forward scattering measurements $\sigma \to \pi$, $\sigma \to \sigma$, $\sigma \to +45°$ and $\sigma \to -45°$.

In the fourth, the main, experiment, the α–^{57}Fe sample and the SS sample A were placed in heterodyne setup, sample A put on the Mössbauer drive. The obtained 2D intensity patterns are displayed in Fig. 2 of the main text. The additional figures Figs. S4–S6 show the results of the stroboscopic evaluation.

We can observe that the $\sigma \to \pi$ scattering channel exhibits no stroboscopic resonances, in agreement with the orthogonality property found in Sect. S4. Also notably, with the calibration parameters and those obtained from the fit of the nuclear resonance forward scattering data (step three), all the spectra could be simulated reliably.

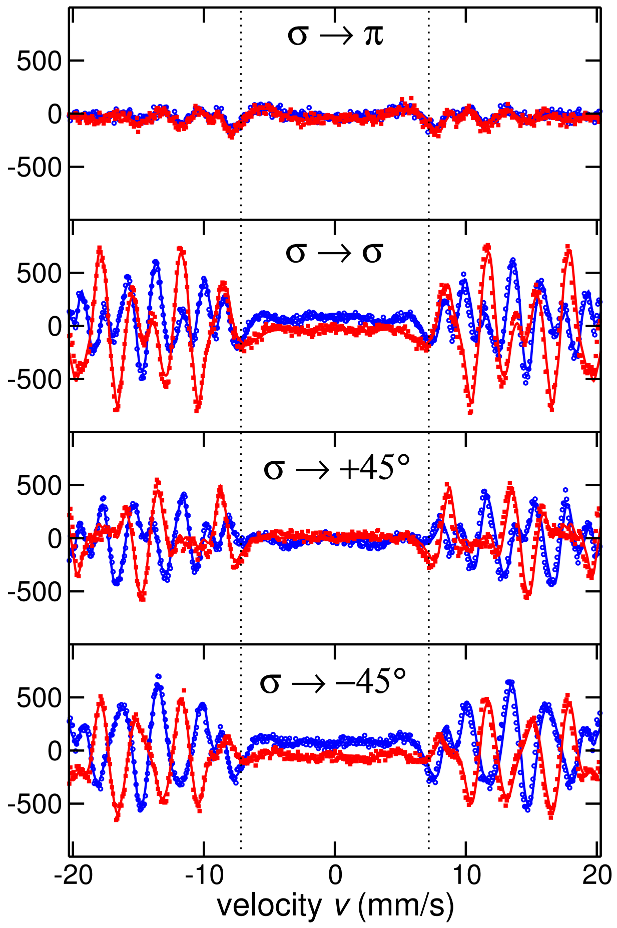

Figure S5: Imaginary part stroboscopic evaluation of direct (red) and reciprocal (blue) scatterings on the α–^{57}Fe foil. Solid lines denote the simultaneous fits on the four nuclear resonance forward scattering measurements $\sigma \to \pi$, $\sigma \to \sigma$, $\sigma \to +45°$ and $\sigma \to -45°$.

We remark that the relative normalization coefficients mentioned at the end of Sect. S4 all turned out to be close to 1. The values were $\lambda^{(\sigma\to+45°)} = 0.712$, $\lambda^{(\sigma\to-45°)} = 1.237$ and $\lambda^{(\sigma\to\pi)} = 0.896$ in the direct case, and $\lambda^{(\sigma\to+45°)} = 0.825$, $\lambda^{(\sigma\to-45°)} = 0.998$ and $\lambda^{(\sigma\to\pi)} = 0.821$ in the reciprocal case.

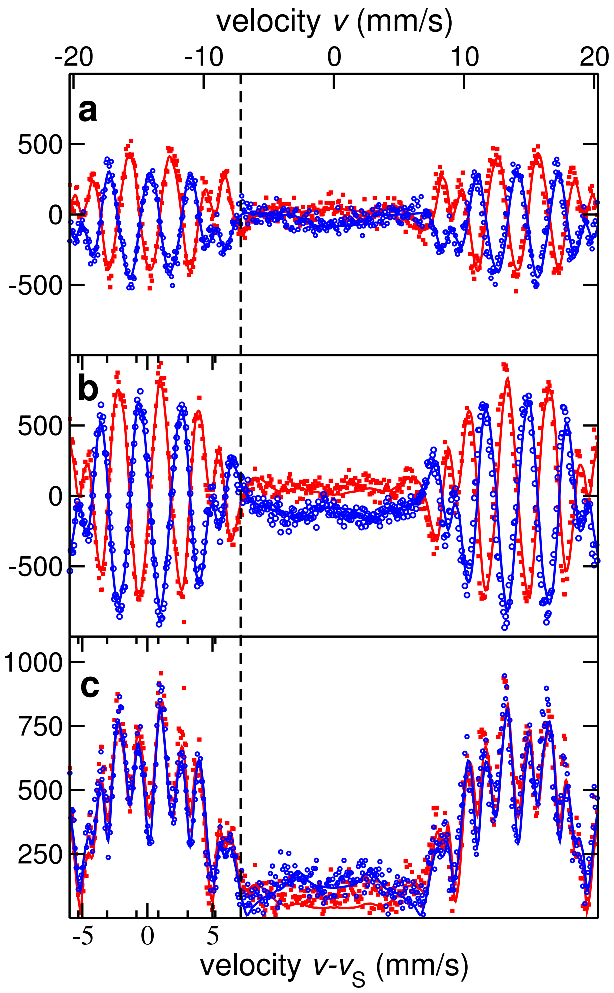

Figure S6: Real part **(a)**, imaginary part **(b)** and magnitude **(c)** of the complex scattering amplitude. Dots are experimental signals and continuous lines are theory simulations. The agreement between direct (red) and reciprocal (blue) signals visible in **(c)** demonstrates magnitude reciprocity, while the opposite signs between direct and reciprocal signals observable in both plots **(a)** and **(b)** prove maximal reciprocity violation in the phase (180° phase difference). The left one-third domain has been used for preparing Fig. 3 of the main text (region indicated by dashed line).
